# Future of Artificial Intelligence Applications in Cancer Care: A Global Cross-Sectional Survey of Researchers

**DOI:** 10.3390/curroncol30030260

**Published:** 2023-03-16

**Authors:** Bernardo Pereira Cabral, Luiza Amara Maciel Braga, Shabbir Syed-Abdul, Fabio Batista Mota

**Affiliations:** 1 Department of Economics, Federal University of Bahia, Salvador 40060-300, Brazil; 2 Laboratory of Cellular Communication, Oswaldo Cruz Institute, Oswaldo Cruz Foundation, Rio de Janeiro 21040-360, Brazil; 3 Graduate Institute of Biomedical Informatics, College of Medical Science and Technology, Taipei Medical University, Taipei 110, Taiwan; 4 School of Gerontology and Long-Term Care, College of Nursing, Taipei Medical University, Taipei 110, Taiwan

**Keywords:** cancer, artificial intelligence, survey research, researcher opinion

## Abstract

Cancer significantly contributes to global mortality, with 9.3 million annual deaths. To alleviate this burden, the utilization of artificial intelligence (AI) applications has been proposed in various domains of oncology. However, the potential applications of AI and the barriers to its widespread adoption remain unclear. This study aimed to address this gap by conducting a cross-sectional, global, web-based survey of over 1000 AI and cancer researchers. The results indicated that most respondents believed AI would positively impact cancer grading and classification, follow-up services, and diagnostic accuracy. Despite these benefits, several limitations were identified, including difficulties incorporating AI into clinical practice and the lack of standardization in cancer health data. These limitations pose significant challenges, particularly regarding testing, validation, certification, and auditing AI algorithms and systems. The results of this study provide valuable insights for informed decision-making for stakeholders involved in AI and cancer research and development, including individual researchers and research funding agencies.

## 1. Introduction

Cancer is one of the leading causes of death in every country globally [1]. World Health Organization (WHO) data for the year 2022 show that cancer is the second leading cause of death among noncommunicable diseases, resulting in 9.3 million deaths per year, second only to cardiovascular diseases (17.9 million) [2]. This prominence as a leading cause of death places cancer as the target of major innovative efforts by academia and the pharmaceutical industry. These efforts have brought several new diagnostic and treatment technologies to the market, such as immunotherapy [3,4] and precision medicine [5]. More recently, great interest has been observed in the search for new diagnostics and treatments involving artificial intelligence (AI) [6,7,8,9,10]. AI is a broad field that comprises various technologies such as deep learning, machine learning, natural language processing, neural networks, and rule-based systems [11,12].

As is the case in many other fields of healthcare, the integration of AI in cancer care is expected to reshape the existing scenario in the future [10]. For example, as a predictive modeling and early detection, AI could be used to analyze data from a variety of sources, such as electronic health records, genetic information, and environmental data, to predict an individual’s risk of developing cancer and to tailor prevention strategies accordingly [13,14,15,16]. AI-related applications may reduce screening costs [17], provide more reliable diagnostics [13,18,19,20], improve prognostics [13,19,21,22,23,24,25], and aid in the discovery of new drugs [14,15]. Several areas of cancer care are expected to benefit from AI-related applications, including cancer radiology and clinical oncology [10]. In the United States alone, more than 70 AI-related applications for different specialties and tumors had received approval from the Food and Drug Administration (FDA) by 2021 [10].

However, different inhibiting factors may affect the creation and adoption of these AI-related applications in cancer care. It may be hampered by ethical and regulatory issues associated with legal uncertainty about responsibility and accountability for AI-supported decisions [10,16,26,27,28,29] or the lack of improvements in medical applications [10,16,26,27,30,31]. The difficulty of incorporation into clinical practice itself [10,16,19,26,27,30,32] and the lack of standardization in cancer-related health data [10,16,19,26,27] may also hamper these new developments.

While the potential applications of AI in cancer care are promising, the barriers to their widespread adoption, such as those discussed, create uncertainty around the success of these technologies in the fight against cancer. This study aims to provide a glimpse into the future of AI in cancer care by gathering the perspectives of researchers involved in this field. To do so, we conducted a cross-sectional survey of authors of recent peer-reviewed articles related to cancer and AI retrieved from the Web of Science Core Collection (WoS).

Previous studies sought to anticipate the future of AI use in cancer care through a literature review [6,9,10,25,33,34,35,36]. Overall, most of them focused on specific areas, such as precision medicine [6], clinical oncology [37], diagnosis [33], and cancer target identification [15]. Other studies focused on broader aspects of cancer care, such as current applications and future perspectives [9,10]. Although our study also conducted a literature review, it did so mainly to identify the relevant aspects posed for the future of the topic and, thus, to design the survey questionnaire applied to the authors above. More comprehensive in scope, our study differs from the others in its method (survey research) and in presenting a common vision about the future of AI in cancer care based on the expectations of more than a thousand researchers in the field. According to the expectations of the survey participants, in this study, we present (a) the likelihood of the occurrence of future events pointed out in the scientific literature (such as reducing screening costs and improving diagnostics), (b) the AI applications most likely to be successful in the future (cancer diagnostics and early cancer detection, for example), (c) the areas of cancer care that are most likely to benefit from AI in the future (e.g., pathology and cancer radiology), and (d) the factors most likely to hamper the use of AI applications in cancer care in the future (such as incorporating AI applications into clinical practice and ethical or regulatory issues).

## 2. Materials and Methods

### 2.1. Literature Review and Questionnaire

We conducted a literature review to identify pressing issues of future AI applications in cancer care. To do so, we selected review articles recently published in WoS-indexed journals. The identification of publications was made with the following query:

TI = ((“Artificial Intelligence” OR “Computational Intelligence” OR “Machine Intelligence” OR “Computer Reasoning” OR “Computer Vision System*” OR “Machine learning” OR “Transfer Learning” OR “Deep Learning” OR “Hierarchical Learning”) AND (Tumor OR Tumors OR Neoplasm OR Neoplasms OR Neoplasia OR Neoplasias OR Cancer OR Cancers))

Timespan: 20 September 2020 to 20 September 2022 (Index Date)

SCI-EXPANDED

Document Types: Review Article

Languages: English

The search strategy combined thesaurus terms related to AI and cancer collected in the Medical Subject Headings (MeSH), US National Library of Medicine (https://www.ncbi.nlm.nih.gov/mesh, acessed on 10 September 2022). In WoS advanced search mode, we used the tag Title (TI) to search for these terms in the titles of review articles published in the last two years (20 September 2020, to 20 September 2022). We used the Science Citation Index Expanded (SCI-EXPANDED) to retrieve only documents published in journals of science. Only review articles written in English were included in the literature review.

The search was done on 20 September 2022, and retrieved 274 publication records, which were imported in plain text format into VantagePoint 11.0 data/text mining software. After reading their titles and abstracts, we selected 74 publication records for further analysis. The records were then imported into Citavi 6.1 reference management software, where we performed the literature review and managed the references. We then downloaded the complete review articles in PDF format, which were entirely read. Of the 74 review articles read, 38 were selected for the literature review and preparation of the survey questionnaire.

The questionnaire considered a 10-year horizon (2022–2032) and was divided into five parts. Initially, we introduced the survey with information about the purpose of the study and aspects related to voluntary participation, absence of sensitive questions, data collection and treatment, and anonymization of results. In addition, the respondents were asked whether they consented to participate in the study—if so, they continued with the questionnaire, and if not, the questionnaire was terminated. Thus, all respondents who participated in this survey gave us their informed consent to use the data collected for research purposes. In the second part, the respondents’ level of knowledge about AI applications in cancer care was asked. Respondents who self-reported having high, good, or some knowledge were qualified for the survey and followed up, while those who reported having no knowledge were disqualified and did not answer the questionnaire.

The third part asked about the likelihood of occurrence of different AI developments in cancer care: (i) whether it would be widely used, (ii) more reliable diagnostics, (iii) reduce screening cost, (iv) improve follow-up services, (v) aid the discovery of new drugs, (vi) grade and classify cancer, and (vii) improve prognostics. The third part also asked the respondents to rank different applications of AI in cancer care, considering their likelihood of success in the next ten years, as well as for them to report—considering the recent FDA approval of artificial intelligence applications in cancer care and their prospects [10]—which specific area of interest would benefit the most from AI use in cancer care.

The fourth part of the questionnaire had two questions regarding general barriers to using AI in cancer care. Respondents could select, among five options (including others), the one they considered most important (e.g., ethical and regulatory issues) and, in sequence, select the most important specific barrier from the option they selected previously (e.g., algorithmic bias). The bibliographic references for each question in the questionnaire are listed in Table 1.

Finally, the fifth and sixth parts of the questionnaire were optional and were not included in the calculation of fully answered questionnaires. The fifth part consisted of an open-ended question, where the respondents were invited to leave comments, suggestions, and criticisms on the questionnaire. The last part covered five demographic questions, where the respondents could report their academic degree, professional occupation, institutional affiliation, professional experience, and region where they live. The demographics of the respondents do not influence the results of this type of study [51,52,53,54]. They were used to present an overview of the study participants.

### 2.2. Survey Respondents

The survey respondents were authors of articles or review articles on AI and cancer published between 20 September 2020 and 20 September 2022) and indexed in WoS SCI-EXPANDED. We used the same search strategy of the literature review, but with two changes: (1) instead of the tag Title, we used the tag Topic (TS), which, besides the title, searches for records in the abstract and keywords; and (2) we added the document type articles. The search was conducted on 20 September 2022, and retrieved 15,533 publication records. We imported these records into VantagePoint 11.0, where we retrieved 28,263 author emails, excluding duplicates. We then created a CSV file with author data (email, name, and article title) and used an in-house developed python code to link 84% (23,740) of these emails to their owners—which allowed us to send personalized emails to most respondents.

### 2.3. Survey Procedures, Ethical Aspects, and Limitations of the Study

The list of respondents with linked and unlinked emails was imported into the SurveyMonkey online survey platform, where the questionnaire was designed, and the survey was conducted. After uploading, the number of emails was reduced to 25,000 due to 2726 bounced emails and 537 opted-out contacts (people who previously opted out of surveys conducted through SurveyMonkey).

Before conducting the formal study, we validated the questionnaire through a pilot with a random sample of 1000 researchers (3.54% of total respondents).

The questionnaire was available for eight consecutive days after the invitation email was sent, and up to three reminder emails were sent to non-responders. In both the invitation and reminder emails, and on the first page of the questionnaire, respondents were informed: (i) of the purpose of the study, (ii) that sensitive data would not be asked, (iii) that the data collected would be anonymized in the results, (iv) that participation would be voluntary, (v) and that informed consent for participation in the study would be sought.

In the pilot, we evaluated the questionnaire (application routine, consistency, internal logic, completion rate, response time, etc.) and allowed the respondents to make observations, suggestions, and criticisms. The 11 respondents who answered the questionnaire did not suggest changes. Then, neither the questionnaire nor the survey procedures were changed in the formal study, and the answers collected in the pilot were included in the study’s results. The pilot was conducted between 16 and 23 October, and the formal study between 24 October and 4 November 2022.

Given that the only personal data of the participants (name and email) were obtained from article records made available in a database of scientific publications (WoS), and considering voluntary participation, absence of sensitive questions, anonymization of results, and obtaining informed consent, examination of the study by an ethics committee was not necessary. In addition, this study followed the Brazilian Resolution number 510 of 7 April 2016 (Official Federal Gazette: https://www.in.gov.br/materia/-/asset_publisher/Kujrw0TZC2Mb/content/id/22917581 (accessed on 10 September 2022)), which exempts from registration and evaluation by an ethics committee public opinion research with unidentified participants.

The procedures adopted in this study followed previous studies that have surveyed researchers and sought to anticipate future possibilities in science and technology [51,52,53,54]. Furthermore, just like them, it shares the same limitations. One of them is the limited diversity of respondents, which is a consequence of the method of identification and selection of respondents in scientific articles. Thus, if respondents are authors of scientific articles, naturally, they will be predominantly composed of researchers and professors linked to universities and research organizations. Another limitation is related to the possibility of respondents’ optimism bias. As they are authors of articles related to AI and cancer—and thus invested in this subject—they may be more optimistic about the future of the technology than other respondent profiles (patients, managers, business people, and politicians, for example). Because they are invested in developing scientific and technological knowledge, they are naturally among the most qualified to inform about future possibilities of AI use in cancer care. As the future of AI uses in cancer care is still quite uncertain, weighing the opinions of researchers on the topic against those of other respondent profiles (with less scientific and technological knowledge) does not seem methodologically relevant to the study.

A final limitation refers to the self-attribution of knowledge level by the respondents in the questionnaire. Unfortunately, it is not possible for the authors of this study to assign levels of knowledge to each respondent nor to assess whether the self-assigned level of knowledge is coherent. Thus, the self-assigned level of knowledge is a function of how the respondents assess their knowledge in the area. In any case, all participants in this study are authors of peer-reviewed scientific articles related to AI and cancer indexed in WoS, reducing the chances of including opinions from people without knowledge of the subject.

### 2.4. Statistical Analysis of the Sample

To verify the statistical differences between the responses of different knowledge levels, we performed analyses in the software IBM-SPSS Statistics 28. To select the appropriate analysis method, we used the Kolmogorov–Smirnov and Shapiro–Wilk tests to check the distribution of the sample. These two tests are the most widely used to check the normality of the sample [55]. The null hypothesis of both is that the data distribution is normal. Its rejection implies that the data do not have a normal distribution. The results of these two tests indicated that the distribution of responses from different levels of knowledge is not normal. Thus, we used the Kruskal–Wallis non-parametric test to check for possible differences between the groups of respondents. This is the non-parametric test recommended for testing differences between more than two group samples from the same population. This test verifies if the groups’ responses are so different that they cannot be considered to belong to the same population. Its null hypothesis is no difference between the groups’ responses [56]. The results of the Kruskal–Wallis test indicated that the responses of the three groups of respondents were not statistically different. Therefore, the responses of the three groups were reported in aggregate. All statistics results are depicted in the Supplementary Material.

## 3. Results

From 25,000 invited researchers, 1030 agreed to participate in the study—after excluding 66 respondents who did not consent to participate. This number gave us a response rate of 4.12%, which is compatible to the response rate found in other future-oriented studies that used the same survey method [52,53,57,58]. Of these 1030 researchers, 26.02% reported having a high knowledge of AI in cancer care, while 42.72% and 28.93% reported having good and some knowledge, respectively. Only 2.33% self-reported having no knowledge of the subject. They were disqualified from the survey and thus did not answer the questionnaire. Of the 1006 questionnaires from high, good, and some knowledge respondents, 881 were completely filled (87.57% of total valid responses). Considering the invited researchers as the survey population (25,000), the minimum required sample size to obtain a 5% margin of error with a confidence level of 95% was 394 completely filled questionnaires. Our 881 completed filled questionnaires gave us a representative sample with a 95% confidence level and a margin of error of 3%, which was high enough to generate consistent results.

**Figure 1:**
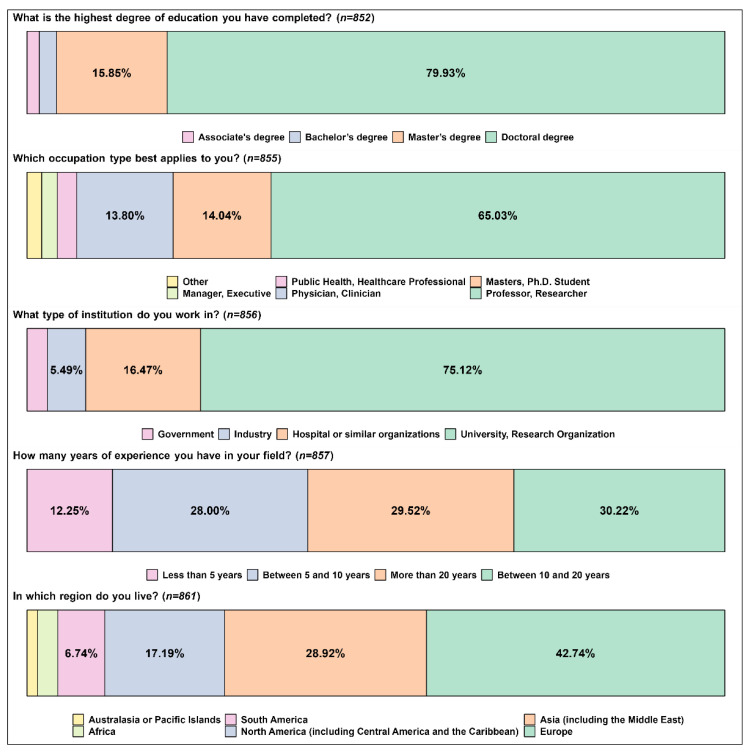
Demographic profile of the respondents.

The demographics of the respondents are depicted in Figure 1. Most had a Doctoral degree (79.93%), while 15.85% had a Master’s degree. As for their occupation, the majority were professors or researchers (65.03%), followed by physicians/clinicians, and Doctoral and Master’s students, with approximately 14% each. Most respondents worked in universities or research organizations (75.12%) and 16.47% in hospitals or similar organizations. Concerning the length of experience, there was a similar distribution among respondents with experience between 5 and 10 years, between 10 and 20 years, and with more than 20 years—approximately 30% each. As for their location, 42.47% lived in Europe, 28.92% in Asia, and 17.19% in North America (including Central America and the Caribbean).

**Figure 2:**
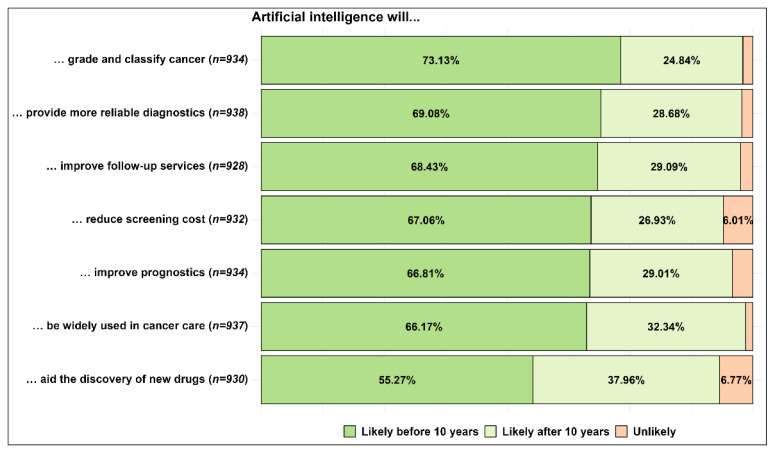
Likelihood of expected future events derived from the use of AI in cancer care.

Figure 2 shows the likelihood of seven different future events derived from the expected use of AI in cancer care. More than 50% of the respondents expected all seven events to occur before ten years. AI grading and classifying cancer was the event that most respondents considered likely in this timespan (73.13%), followed by providing more reliable diagnoses (69.08%). In turn, aiding in discovering new drugs and being widely used in cancer care obtained the highest likely percentages after ten years (37.96% and 32.34%, respectively). The unlikely percentages were low, with the highest also for AI aiding new drug discovery (6.77%).

**Figure 3:**
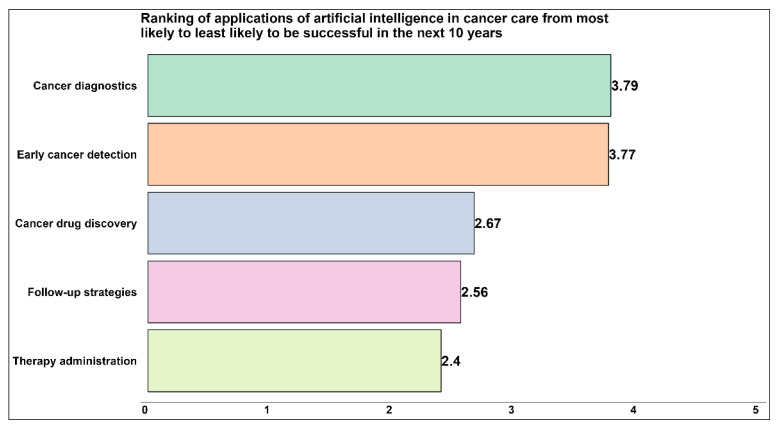
Average ranking of AI applications most likely to be successful in cancer care in the next ten years. Ranking on a 5-point scale, where 1 is the most likely and 5 is the least likely. SurveyMonkey calculated the ranking result. Applying weights in reverse, SurveyMonkey calculates the average ranking for each respondent’s choice to determine the most preferred choice overall (SurveyMonkey: https://help.surveymonkey.com/en/surveymonkey/create/ranking-question/, accessed on 20 November 2022).

Figure 3 depicts the average ranking of AI applications considered most likely to be successful in cancer care in the next ten years. The respondents’ most preferred application was cancer diagnostics (3.79), followed closely by early cancer detection (3.77). Therapy administration was the least preferred application to be successful in this timespan (2.4).

**Figure 4:**
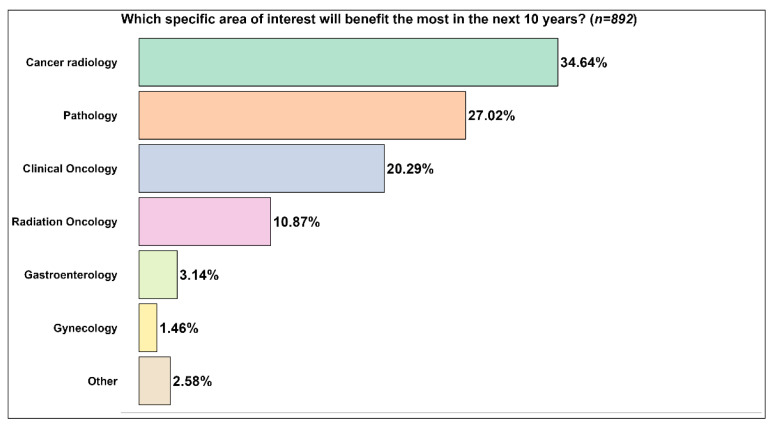
Areas of interest most likely to benefit from AI in the next ten years.

Considering AI applications in cancer care recently approved by the FDA [10], the respondents indicated the areas of interest [10] that would benefit most from the use of AI in the next ten years (Figure 4). For about one-third of them, cancer radiology would benefit the most, followed by pathology (27.02%). Of the available options, gynecology was considered to benefit the least from using AI (1.46%). A small part of the respondents (2.58%) pointed out that other areas not listed in the questionnaire would benefit the most. A small part of them (2.58%) pointed out that other areas not listed in the questionnaire would benefit the most.

**Figure 5:**
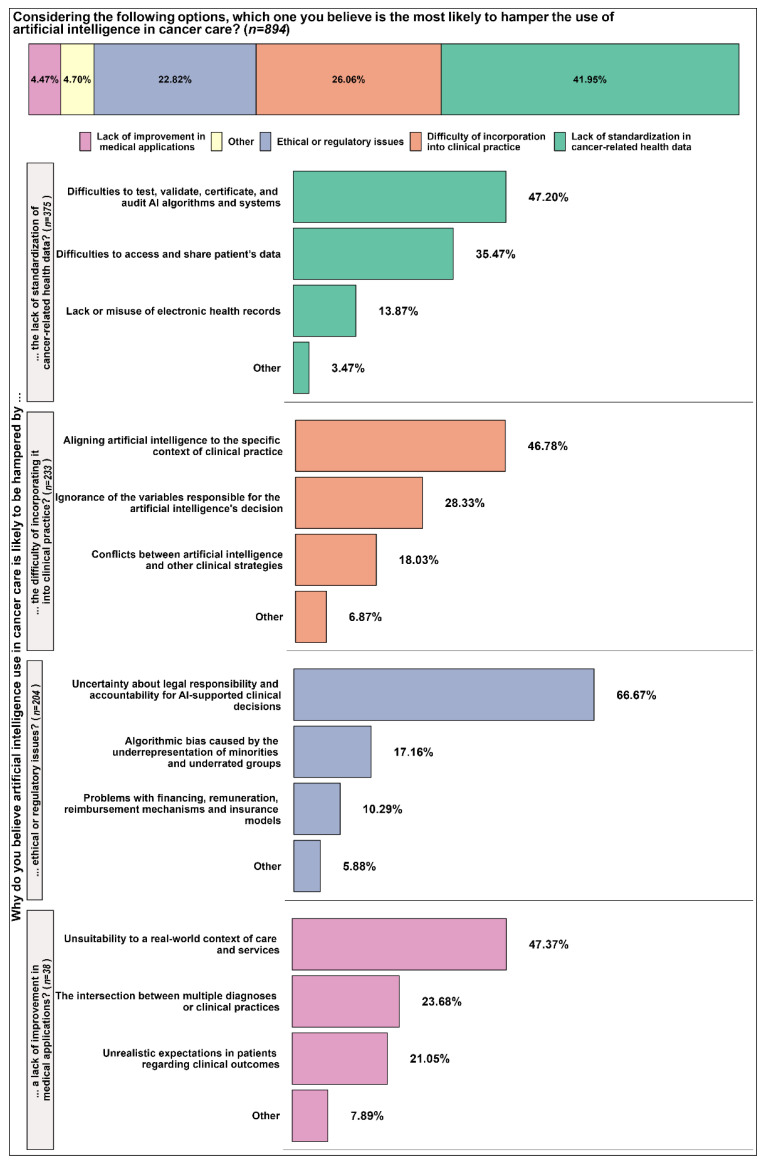
Barriers to the use of AI in cancer care.

The lack of standardization in cancer-related health data was considered the most likely barrier to AI use in cancer care (Figure 5). It was the choice of 41.95% of respondents, followed by the difficulty of incorporation into clinical practice (26.06%) and ethical or regulatory issues (22.82%). The lack of improvement in medical applications and other barriers amounted to less than 10% of total responses (894). Of the 375 respondents who selected the lack of standardization in cancer-related health data, 47.20% believed that the main reason for the lack of standardization originated from difficulties in testing, validating, certifying, and auditing AI algorithms and systems. Another 35.47% attributed the lack of standardization to difficulties accessing and sharing patient data. Among the 233 respondents who chose the difficulty of incorporating AI into clinical practice, 46.78% attributed their choice to the difficulty of aligning AI to the specific context of clinical practice. Finally, among those who chose ethical or regulatory issues (204), 66.67% believed that the use of AI in cancer care was likely to be hampered by uncertainties about legal responsibility and accountability for AI-supported clinical decisions.

## 4. Discussion

The responses from 1006 authors of articles (experts) on AI and cancer provided us with an in-depth understanding of AI’s potential opportunities and challenges in cancer care. Aligning with the number of SaMD (Software as Medical Devices) recently approved by the US-FDA [10], a significant proportion of respondents believed that cancer radiology (34.64%) and pathology (27.02%) would be the areas that would benefit most from the use of AI in cancer care over the next decade. In turn, only 1.46% chose gynecology. Some possible reasons could be the complexity of the field, which involves not only diagnosis and treatment, but also a wide range of mother and child conditions and responses to the treatment. It requires intuitive decision-making from the consultant. Another reason could be the lack of data to train AI models. Gynecologists may be skeptical about AI’s reliability and effectiveness in real-time medical practice, which could limit its adoption.

Interestingly, this study’s results align with reports that gynecology is the least SaMD-approved field by the FDA [10]. On the other hand, an increasing number of studies report using AI to evaluate images such as MRI, colposcopy, fetal ultrasound [59], and hysteroscopy [60]. If we had asked for gynecology–radiology, perhaps we would have had more respondents choosing it.

One of the major areas where AI could have a significant impact is early cancer detection [20,48,49]. This was one of the most preferred applications of AI in cancer care by the respondents of this survey. Currently, many cancer cases are not diagnosed until the disease has advanced, making treatment more difficult and less successful. Several large-scale studies have reported that using AI to analyze lung CT images for lung cancer screening confirms survival benefits [61] and that it helps with the precise diagnosis and treatment of liver and brain cancers [62,63]. By analyzing medical images and other data, AI algorithms can help identify signs of cancer at an early stage, increasing the chances of successful treatment. For example, AI can analyze mammograms to identify breast cancer, CT scans to identify lung cancer, and polyps indicative of colorectal cancer in real-time [18,20,48,49,50,64,65]. AI can also help with early detection by analyzing a patient’s medical history [5] and test results to identify patterns that may indicate the presence of cancer [47]. For example, AI algorithms can analyze genetic data to identify mutations associated with increased cancer risk. By analyzing this data, doctors can determine the most appropriate course of treatment for each patient [10,16,22].

When asked about the likelihood of future AI applications in cancer care, 73.13% of respondents selected grading and classifying the cancer stages, which means image analysis, followed by 69.08% of respondents who thought AI would be useful to provide a more reliable diagnosis within the next ten years. For example, Watson analyzed a patient’s medical record and generated recommendations for treatment options by selecting from a list of possibilities, scoring their appropriateness for the patient on a percentage scale, and presenting them to the clinician for consideration [66]. Another area where AI could majorly impact is the discovery of new drugs. This expectation was shared by most of the respondents in this study. By analyzing data from clinical trials and other sources, AI algorithms can identify patterns that may indicate the potential effectiveness of a new drug [14,15]. This could help speed up the drug development process, potentially leading to new treatments that are more effective and have fewer side effects.

When we surveyed the factors hampering the use of AI in cancer care, uncertainty about legal responsibility and accountability for AI-supported clinical decisions was the choice of most (66.67%) of the 22.82% of respondents who selected ethical or regulatory issues. To address the uncertainty, it may be necessary to establish clear guidelines and regulations around the use of AI in clinical practice, including standards for data collection, storage, and use, as well as guidelines for transparency and accountability in decision-making processes [66]. The explainability of AI models is gaining importance in clinical practices. Transparent algorithms or explanatory approaches create trust and can make adopting AI systems less risky for clinical practitioners [67].

Another important factor that must be considered is AI’s ethical implications in cancer care, including issues of bias. As it is known, one potential issue with the use of AI in cancer care is the risk of bias in the algorithms used [30,68]. About 17% of the 22.82% of the respondents who chose ethical or regulatory issues believed algorithmic bias caused by the underrepresentation of minorities and underrated groups was the most likely factor to hamper Ai use in cancer care. If the data used to train the algorithms were not representative of the treated population, the AI may not be able to diagnose or treat certain groups of patients accurately. For example, if the data used to train an AI algorithm to detect breast cancer are predominantly from white women, the algorithm may not be as effective at detecting breast cancer in women of other races [69]. Thus, one of the biggest challenges is the generalizability of AI algorithms. To address this issue, ensuring that the data used to train AI algorithms are diverse and representative of the population being treated is important. To create more reliable, accurate, and generalizable AI models, it is necessary to have a deeper understanding of the ethical considerations surrounding the use of AI, including how to interpret its performance, standardize techniques, and identify and address bias [69]. To ensure that the AI is trained accurately and effectively, it is necessary to include a diverse range of individuals in terms of ethnicity, age, and sex, as well as examples of benign and malignant tumors. Additionally, when implementing precision medicine and AI in real-world clinical settings, it is important to consider environmental factors, challenges related to providing care in resource-poor areas, and multiple concurrent medical conditions [70].

The difficulty of incorporating AI into clinical practices was another important hampering factor, as 26.06% of the respondents reported it could hamper AI use in cancer care in the Future. This would be mainly due to issues regarding the alignment of AI to the specific context of clinical practice, according to 46.78% of those respondents. One challenge in integrating AI with clinical practice is the generalizability and reproducibility of AI algorithms. This is because, in clinical practice, machine learning models may encounter real-world data that are incomplete or contains errors, despite being trained on datasets that have been carefully cleaned to eliminate poor-quality information [71].

Many respondents who reported that the lack of standardization in cancer-related health data was the most likely factor to hamper AI use in cancer care believed this would be due to difficulties in testing, validating, certifying, and auditing AI algorithms and systems (47.20%). Still, regarding the lack of standardization, the lack or misuse of electronic health records was considered by fewer respondents (13.87%) as likely to hamper the use of AI in cancer care. With the increasing use of electronic health records, there was a risk that patient data could be accessed or shared without the patient’s knowledge or consent [72]. Therefore, it is important to ensure appropriate safeguards to protect patient privacy and prevent data misuse. Additionally, challenges such as data breaches, ransomware attacks, and hackers have hampered the adoption process among healthcare providers [73].

## 5. Conclusions

This study presents a comprehensive evaluation of the views and expectations of 1030 experts in the field of AI applications in cancer. Through a cross-sectional, global, web-based survey, the researchers were asked about their views on the future of AI in cancer care. The results indicated that most respondents believed AI would play a critical role in cancer prediction, early detection, grading, and classification, thus improving follow-up services and providing more reliable diagnostics.

Despite these benefits, incorporating AI into clinical practice may be challenging due to the lack of standardization in cancer-related health data. Specifically, these limitations may hinder the testing, validation, certification, and auditing of AI algorithms and systems. The results of this study provide valuable insights into the future trends and potential of AI in cancer care and can inform the research and development decisions of various stakeholders, including individual researchers and research funding agencies, both public and private.

In conclusion, this study highlights the importance of addressing the barriers to the widespread adoption of AI in cancer care to fully realize the potential of these technologies in improving patient outcomes and reducing the burden of cancer worldwide.

## Figures and Tables

**Table 1:** References used in the development of the survey questionnaire.

Topic	Alternatives	References
Broad use of artificial intelligence in the future	Likely before or after ten years; unlikely	[7,10,13,19,34,35]
Possible artificial intelligence applications in cancer care in the future	More reliable diagnostics	[8,13,18,19,23,25,36,38]
	Reduce screening cost	[20,21,34,39,40,41,42,43]
	Improve follow-up services	[9,10,37]
	Discovery of new drugs	[9,10,14,15]
	Grade and classify cancer	[22,30,44,45,46]
	Improve prognostics	[19,22,24,36,47]
Possible applications of artificial intelligence in cancer care	Drug discovery	[14,15]
	Early detection	[20,48,49,50]
	Diagnostics	[8,13,18,19,22,24,25,33,36,42]
	Therapy administration	[7,15,19,34]
	Follow-up strategies	[6,7,10,19]
Specific areas of interest for future developments of artificial intelligence applications in cancer care	Pathology, clinical oncology, radiation oncology, gastroenterology, gynecology	[10]
Factors hampering artificial intelligence adoption in cancer care	The difficulty of incorporation into clinical practice	[10,16,19,26,27,30,32]
	Ethical or regulatory issues	[10,16,26,27,28,29]
	Lack of improvement in medical applications	[10,16,26,27,30,31]
	Lack of standardization in cancer-related health data	[10,16,19,26,27]

## Data Availability

The data presented in this study are available as Supplementary Material.

## Supplementary Materials

The following supporting information can be downloaded at: https://www.mdpi.com/article/10.3390/curroncol30030260/s1.
