# Supplementary material for: Future of Artificial Intelligence Applications in Cancer Care: A Global Cross-Sectional Survey of Researchers
Source: Curr Oncol. 2023 Mar 16;30(3):3432–46. doi: 10.3390/curroncol30030260 (PMC10047823; doi:10.3390/curroncol30030260)
Supplement: Supplementary file 1 [file curroncol-30-00260-s001.zip › curroncol-2249364-supplementary.pdf]

Supplementary Material – Statistical Analysis (Knowledge level)

Please indicate your knowledge level on artificial intelligence in cancer care

**Sample size**		942	
**Margin of error**		3.20%	
**Mean**		2.02	
**Standard deviation**		0.746	
**Percentiles**	¼	1 (High knowledge)	
	½	2 (Good knowledge)	
	¾	3 (Some knowledge)	
**Kolmogorov-Smirnov**		**Test ratio**	**P-value**
		0.223	0.000
**Shapiro-Wilk**		**Test ratio**	**P-value**
		0.809	0.000

	**Some knowledge**	**Good knowledge**	**High knowledge**	**Total**
**Number of observations**	272	418	252	942
**Frequency**	28.87%	44.37%	26.75%	100%

Artificial intelligence will be widely used in cancer care

**Sample size**		937	
**Margin of error**		3.21%	
**Mean**		1.35	
**Standard deviation**		0.509	
**Percentiles**	¼	1 (Likely before 10 years)	
	½	1 (Likely before 10 years)	
	¾	2 (Likely after 10 years)	
**Kruskal-Wallis**		**Statistic**	**P-value**
		1.592	0.451
**Kolmogorov-Smirnov**		**Statistic**	**P-value**
		0.418	0.000
**Shapiro-Wilk**		**Statistic**	**P-value**
		0.630	0.000

		**Unlikely**	**Likely after 10 years**	**Likely before 10 years**	**Total**
**Some knowledge**	**Number of observations**	1	93	175	269
	**Frequency**	0.37%	34.57%	65.06%	100.00%
**Good knowledge**	**Number of observations**	7	140	270	417
	**Frequency**	1.68%	33.57%	64.75%	100.00%
**High Knowledge**	**Number of observations**	6	70	175	251
	**Frequency**	2.39%	27.89%	69.72%	100.00%

Artificial intelligence will provide more reliable diagnostics

**Sample size**		938	
**Margin of error**		3.21%	
**Mean**		1.33	
**Standard deviation**		0.516	
**Percentiles**	¼	1 (Likely before 10 years)	
	½	1 (Likely before 10 years)	
	¾	2 (Likely after 10 years)	
**Kruskal-Wallis**		**Statistic**	**P-value**
		1.729	0.421
**Kolmogorov-Smirnov**		**Statistic**	**P-value**
		0.430	0.000
**Shapiro-Wilk**		**Statistic**	**P-value**
		0.618	0.000

		**Unlikely**	**Likely after 10 years**	**Likely before 10 years**	**Total**
**Some knowledge**	**Number of observations**	6	80	184	270
	**Frequency**	2.22%	29.63%	68.15%	100.00%
**Good knowledge**	**Number of observations**	12	122	283	417
	**Frequency**	2.88%	29.26%	67.87%	100.00%
**High Knowledge**	**Number of observations**	3	67	181	251
	**Frequency**	1.20%	26.69%	72.11%	100.00%

Artificial intelligence will reduce screening cost

**Sample size**		932	
**Margin of error**		3.21%	
**Mean**		1.39	
**Standard deviation**		0.599	
**Percentiles**	¼	1 (Likely before 10 years)	
	½	1 (Likely before 10 years)	
	¾	2 (Likely after 10 years)	
**Kruskal-Wallis**		**Statistic**	**P-value**
		2.721	0.257
**Kolmogorov-Smirnov**		**Statistic**	**P-value**
		0.413	0.000
**Shapiro-Wilk**		**Statistic**	**P-value**
		0.647	0.000

		**Unlikely**	**Likely after 10 years**	**Likely before 10 years**	**Total**
**Some knowledge**	**Number of observations**	12	68	189	269
	**Frequency**	4.46%	25.28%	70.26%	100.00%
**Good knowledge**	**Number of observations**	25	122	265	412
	**Frequency**	6.07%	29.61%	64.32%	100.00%

High Knowledge	Number of observations	19	61	171	251
	Frequency	7.57%	24.30%	68.13%	100.00%

Artificial intelligence will improve follow-up services

Sample size		928	
Margin of error		3.22%	
Mean		1.34	
Standard deviation		0.524	
Percentiles	¼	1 (Likely before 10 years)	
	½	1 (Likely before 10 years)	
	¾	2 (Likely after 10 years)	
Kruskal-Wallis		Statistic	P-value
		3.718	0.156
Kolmogorov-Smirnov		Statistic	P-value
		0.426	0.000
Shapiro-Wilk		Statistic	P-value
		0.624	0.000

		Unlikely	Likely after 10 years	Likely before 10 years	Total
Some knowledge	Number of observations	4	79	185	268
	Frequency	1.49%	29.48%	69.03%	100.00%
Good knowledge	Number of observations	14	128	271	413
	Frequency	3.39%	30.99%	65.62%	100.00%
High Knowledge	Number of observations	5	63	179	247
	Frequency	2.02%	25.51%	72.47%	100.00%

Artificial intelligence will aid the discovery of new drugs

Sample size		930	
Margin of error		3.22%	
Mean		1.52	
Standard deviation		0.621	
Percentiles	¼	1 (Likely before 10 years)	
	½	1 (Likely before 10 years)	
	¾	2 (Likely after 10 years)	
Kruskal-Wallis		Statistic	P-value
		5.386	0.068
Kolmogorov-Smirnov		Statistic	P-value
		0.349	0.000
Shapiro-Wilk		Statistic	P-value
		0.719	0.000

		Unlikely	Likely after 10 years	Likely before 10 years	Total
Some knowledge	Number of observations	22	110	136	268
	Frequency	8.21%	41.04%	50.75%	100.00%

Good knowledge	Number of observations	24	163	225	412
	Frequency	5.83%	39.56%	54.61%	100.00%
High Knowledge	Number of observations	17	80	153	268
	Frequency	6.34%	29.85%	57.09%	100.00%

Artificial intelligence will grade and classify cancer

**Sample size**		934	
**Margin of error**		3.21%	
**Mean**		1.29	
**Standard deviation**		0.497	
**Percentiles**	¼	1 (Likely before 10 years)	
	½	1 (Likely before 10 years)	
	¾	2 (Likely after 10 years)	
**Kruskal-Wallis**		**Statistic**	**P-value**
		4.301	0.116
**Kolmogorov-Smirnov**		**Statistic**	**P-value**
		0.451	0.000
**Shapiro-Wilk**		**Statistic**	**P-value**
		0.583	0.000

		Unlikely	Likely after 10 years	Likely before 10 years	Total
Some knowledge	Number of observations	4	73	191	268
	Frequency	1.49%	27.24%	71.27%	100.00%
Good knowledge	Number of observations	11	108	296	415
	Frequency	2.65%	26.02%	71.33%	100.00%
High Knowledge	Number of observations	4	51	196	251
	Frequency	1.59%	20.32%	78.09%	100.00%

Artificial intelligence will improve prognostics

**Sample size**		934	
**Margin of error**		3.21%	
**Mean**		1.37	
**Standard deviation**		0.564	
**Percentiles**	¼	1 (Likely before 10 years)	
	½	1 (Likely before 10 years)	
	¾	2 (Likely after 10 years)	
**Kruskal-Wallis**		**Statistic**	**P-value**
		3.758	0.153
**Kolmogorov-Smirnov**		**Statistic**	**P-value**
		0.414	0.000
**Shapiro-Wilk**		**Statistic**	**P-value**
		0.644	0.000

		Unlikely	Likely after 10 years	Likely before 10 years	Total

Some knowledge	Number of observations	12	85	171	268
	Frequency	4.48%	31.72%	63.81%	100.00%
Good knowledge	Number of observations	20	121	274	415
	Frequency	4.82%	29.16%	66.02%	100.00%
High Knowledge	Number of observations	7	65	179	251
	Frequency	2.79%	25.90%	71.31%	100.00%

Please rank the following applications of artificial intelligence in cancer care from most likely to least likely to be successful in the next 10 years (5 point scale. where 1 is the most likely and 5 is the least likely)

	Most Likely (1)	2	3	4	Least likely (5)
**Cancer drug discovery**	16.27%	12.17%	22.61%	19.75%	29.19%
**Early cancer detection**	38.02%	28.61%	15.31%	9.03%	9.03%
**Cancer diagnostics**	32.68%	36.35%	15.30%	9.06%	6.61%
**Therapy administration**	5.76%	12.48%	21.97%	36.01%	23.77%
**Follow-up strategies**	9.60%	13.11%	27.91%	22.94%	26.44%

		Cancer drug discovery	Early cancer detection	Cancer diagnostics	Therapy administration	Follow-up strategies
**Kruskal-Wallis**	**Statistic**	0.765	1.402	1.353	0.634	0.211
	**P-value**	0.682	0.496	0.508	0.728	0.900

Cancer drug discovery							
		**1**	**2**	**3**	**4**	**5**	**Total**
**Some knowledge**	**Number of observations**	41	26	53	42	76	238
	**Frequency**	17.23%	10.92%	22.27%	17.65%	31.93%	100.00%
**Good knowledge**	**Number of observations**	56	45	72	65	106	344
	**Frequency**	16.28%	13.08%	20.93%	18.90%	30.81%	100.00%
**High Knowledge**	**Number of observations**	34	27	57	52	53	223

	Frequency	15.25%	12.11%	25.56%	23.32%	23.77%	100.00%

Early cancer detection							
		1	2	3	4	5	Total
Some knowledge	Number of observations	86	62	36	33	19	236
	Frequency	36.44%	26.27%	15.25%	13.98%	8.05%	100.00%
Good knowledge	Number of observations	130	99	61	24	27	341
	Frequency	38.12%	29.03%	17.89%	7.04%	7.92%	100.00%
High Knowledge	Number of observations	87	67	25	15	26	220
	Frequency	39.55%	30.45%	11.36%	6.82%	11.82%	100.00%

Cancer diagnostics							
		1	2	3	4	5	Total
Some knowledge	Number of observations	76	85	37	22	18	238
	Frequency	31.93%	35.71%	15.55%	9.24%	7.56%	100.00%
Good knowledge	Number of observations	124	125	62	24	22	357
	Frequency	34.73%	35.01%	17.37%	6.72%	6.16%	100.00%
High Knowledge	Number of observations	67	87	26	28	14	222
	Frequency	30.18%	39.19%	11.71%	12.61%	6.31%	100.00%

Therapy administration							
		1	2	3	4	5	Total
Some knowledge	Number of observations	14	34	54	80	58	240
	Frequency	5.83%	14.17%	22.50%	33.33%	24.17%	100.00%
Good knowledge	Number of observations	124	125	62	24	22	357
	Frequency	34.73%	35.01%	17.37%	6.72%	6.16%	100.00%
High Knowledge	Number of observations	16	22	51	76	60	225
	Frequency	7.11%	9.78%	22.67%	33.78%	26.67%	100.00%

Follow-up strategies							
		1	2	3	4	5	Total
Some knowledge	Number of observations	22	37	71	59	64	253
	Frequency	8.70%	14.62%	28.06%	23.32%	25.30%	100.00%

Good knowledge	**Number of observations**	36	52	106	93	105	392
	**Frequency**	9.18%	13.27%	27.04%	23.72%	26.79%	100.00%
High Knowledge	**Number of observations**	27	27	70	51	65	240
	**Frequency**	11.25%	11.25%	29.17%	21.25%	27.08%	100.00%

Considering the recent FDA approval of artificial intelligence applications in cancer care and their future prospects, in your opinion which specific area of interest will benefit the most in the next 10 years?

**Sample size**		892	
**Margin of error**		3.29%	
**Mean**		3.460	
**Standard deviation**		2.167	
**Percentiles**	¼	1 (Pathology)	
	½	3 (Radiation Oncology)	
	¾	6 (Cancer radiology)	
**Kruskal-Wallis**		**Statistic**	**P-value**
		4.034	0.133
**Kolmogorov-Smirnov**		**Statistic**	**P-value**
		0.252	0.000
**Shapiro-Wilk**		**Statistic**	**P-value**
		0.802	0.000

	**Some knowledge**		**Good knowledge**		**High Knowledge**	
	**Number of observations**	**Frequency**	**Number of observations**	**Frequency**	**Number of observations**	**Frequency**
**Cancer radiology**	84	33.07%	149	37.44%	76	31.67%
**Clinical Oncology**	58	22.83%	83	20.85%	40	16.67%
**Gastroenterology**	8	3.15%	13	3.27%	7	2.92%
**Gynecology**	4	1.57%	7	1.76%	2	0.83%
**Pathology**	66	25.98%	96	24.12%	79	32.92%
**Radiation Oncology**	25	9.84%	41	10.30%	31	12.92%
**Other**	9	3.54%	9	2.26%	5	2.08%
**Total**	254	100.00%	398	100.00%	240	100.00%

Considering the following options, which one you believe is the most likely to hamper the use of artificial intelligence in cancer care?

**Sample size**	894

**Margin of error**		3.28%	
**Mean**		3.275	
**Standard deviation**		1.829	
**Percentiles**	¼	1 (Difficulty of incorporation into clinical practice)	
	½	4 (Lack of improvement in medical applications)	
	¾	5 (Lack of standardization in cancer-related health data)	
**Kruskal-Wallis**		**Statistic**	**P-value**
		0.607	0.738
**Kolmogorov-Smirnov**		**Statistic**	**P-value**
		0.294	0.000
**Shapiro-Wilk**		**Statistic**	**P-value**
		0.784	0.000

	**Some knowledge**		**Good knowledge**		**High Knowledge**	
	**Number of observations**	**Frequency**	**Number of observations**	**Frequency**	**Number of observations**	**Frequency**
**Difficulty of incorporation into clinical practice**	64	25.10%	106	26.57%	63	26.25%
**Ethical or regulatory issues**	50	19.61%	93	23.31%	61	25.42%
**Lack of improvement in medical applications**	14	5.49%	15	3.76%	11	4.58%
**Lack of standardization in cancer-related health data**	120	47.06%	165	41.35%	90	37.50%
**Other**	7	2.75%	20	5.01%	15	6.25%
**Total**	255	100.00%	399	100.00%	240	100.00%

Why do you believe artificial intelligence use in cancer care is likely to be hampered by the difficulty of incorporating it into clinical practice?

**Sample size**		233	
**Margin of error**		6.52%	
**Mean**		2.425	
**Standard deviation**		0.863	
**Percentiles**	¼	2 (Ignorance of the variables responsible for the artificial intelligence's decision)	
	½	3 (Aligning artificial intelligence to the specific context of clinical practice)	
	¾	3 (Aligning artificial intelligence to the specific context of clinical practice)	
**Kruskal-Wallis**		**Statistic**	**P-value**
		0.659	0.719
**Kolmogorov-Smirnov**		**Statistic**	**P-value**
		0.284	0.000
**Shapiro-Wilk**		**Statistic**	**P-value**
		0.845	0.000

	Some knowledge		Good knowledge		High Knowledge	
	Number of observations	Frequency	Number of observations	Frequency	Number of observations	Frequency
**Aligning artificial intelligence to the specific context of clinical practice**	30	46.88%	52	49.06%	27	42.86%
**Conflicts between artificial intelligence and other clinical strategies**	14	21.88%	17	16.04%	11	17.46%
**Ignorance of the variables responsible for the artificial intelligence's decision**	17	26.56%	31	29.25%	18	28.57%
**Other**	3	4.69%	6	5.66%	7	11.11%
**Total**	64	100.00%	106	100.00%	63	100.00%

Why do you believe artificial intelligence use in cancer care is likely to be hampered by ethical or regulatory issues?

**Sample size**		204	
**Margin of error**		6.97%	
**Mean**		1.554	
**Standard deviation**		0.900	
**Percentiles**	¼	1 (Uncertainty about legal responsibility and accountability for AI-supported clinical decisions)	
	½	1 (Uncertainty about legal responsibility and accountability for AI-supported clinical decisions)	
	¾	2 (Algorithmic bias caused by the underrepresentation of minorities and underrated groups)	
**Kruskal-Wallis**		**Statistic**	**P-value**
		3.280	0.194
**Kolmogorov-Smirnov**		**Statistic**	**P-value**
		0.398	0.000
**Shapiro-Wilk**		**Statistic**	**P-value**
		0.654	0.000

	Some knowledge		Good knowledge		High Knowledge	
	Number of observations	Frequency	Number of observations	Frequency	Number of observations	Frequency
Algorithmic bias caused by the underrepresentation of minorities and underrated groups	7	14.00%	22	23.66%	6	9.84%
Problems with financing. remuneration. reimbursement mechanisms and insurance models	4	8.00%	11	11.83%	6	9.84%
Uncertainty about legal responsibility and accountability for AI-supported clinical decisions	37	74.00%	55	59.14%	44	72.13%
Other	2	4.00%	5	5.38%	5	8.20%
Total	50	100.00%	93	100.00%	61	100.00%

Why do you believe artificial intelligence use in cancer care is likely to be hampered by a lack of improvement in medical applications?

Sample size		38	
Margin of error		16.21%	
Mean		2.132	
Standard deviation		0.875	
Percentiles	¼	2 (Unsuitability to a real-world context of care and services)	
	½	2 (Unsuitability to a real-world context of care and services)	
	¾	3 (Unrealistic expectations in patients regarding clinical outcomes)	
Kruskal-Wallis		Statistic	P-value
		0.106	0.948
Kolmogorov-Smirnov		Statistic	P-value
		0.270	0.000
Shapiro-Wilk		Statistic	P-value
		0.857	0.000

	Some knowledge	Good knowledge	High Knowledge

	Number of observations	Frequency	Number of observations	Frequency	Number of observations	Frequency
**The intersection between multiple diagnoses or clinical practices**	3	21.43%	2	15.38%	4	36.36%
**Unrealistic expectations in patients regarding clinical outcomes**	2	14.29%	2	15.38%	4	36.36%
**Unsuitability to a real-world context of care and services**	8	57.14%	8	61.54%	2	18.18%
**Other**	1	7.14%	1	7.69%	1	9.09%
**Total**	14	100.00%	13	100.00%	11	100.00%

Why do you believe artificial intelligence use in cancer care is likely to be hampered by the lack of standardization of cancer-related health data?

**Sample size**		375	
**Margin of error**		5.13%	
**Mean**		2.187	
**Standard deviation**		0.966	
**Percentiles**	¼	1 (Difficulties to access and share patient's data)	
	½	3 (Difficulties to test. validate. certificate. and audit AI algorithms and systems)	
	¾	3 (Difficulties to test. validate. certificate. and audit AI algorithms and systems)	
**Kruskal-Wallis**		**Statistic**	**P-value**
		1.936	0.380
**Kolmogorov-Smirnov**		**Statistic**	**P-value**
		0.307	0.000
**Shapiro-Wilk**		**Statistic**	**P-value**
		0.772	0.000

	Some knowledge		Good knowledge		High Knowledge	
	**Number of observations**	**Frequency**	**Number of observations**	**Frequency**	**Number of observations**	**Frequency**
**Difficulties to access and share patient's data**	35	26.92%	69	40.35%	36	37.50%

**Difficulties to test. validate. certificate. and audit AI algorithms and systems**	69	53.08%	76	44.44%	44	45.83%
**Lack or misuse of electronic health records**	23	17.69%	18	10.53%	13	13.54%
**Other**	3	2.31%	8	4.68%	3	3.13%
**Total**	130	100.00%	171	100.00%	96	100.00%
